# Comparison of cerebral blood flow in oral somatic delusion in patients with and without a history of depression: a comparative case series

**DOI:** 10.1186/s12888-015-0422-0

**Published:** 2015-03-10

**Authors:** Motoko Watanabe, Yojiro Umezaki, Anna Miura, Yukiko Shinohara, Tatsuya Yoshikawa, Tomomi Sakuma, Chisa Shitano, Ayano Katagiri, Miho Takenoshita, Akira Toriihara, Akihito Uezato, Toru Nishikawa, Haruhiko Motomura, Akira Toyofuku

**Affiliations:** 1 Department of Psychosomatic Dentistry, Graduate School of Medical and Dental Sciences, Tokyo Medical and Dental University, 1-5-45 Yushima, Bunkyo-ku, Tokyo 113-8549 Japan; 2 Psychosomatic Dentistry Clinic, Tokyo Medical and Dental University Dental Hospital, 1-5-45 Yushima, Bunkyo-ku, Tokyo 113-8549 Japan; 3 Department of Diagnostic Radiology and Oncology, Graduate School of Medical and Dental Sciences, Tokyo Medical and Dental University, 1-5-45 Yushima, Bunkyo-ku, Tokyo 113-8519 Japan; 4 Department of Psychiatry and Behavioral Sciences, Graduate School of Medical and Dental Sciences, Tokyo Medical and Dental University, 1-5-45 Yushima, Bunkyo-ku, Tokyo 113-8519 Japan

**Keywords:** Brain perfusion, Delusional disorder somatic type, Oral somatic delusion, Remitted depression, SPECT, Temporal region

## Abstract

**Background:**

A significant number of patients visit dental clinics because of unusual oral sensations for which no physical cause can be found. Such patients are recognized as having oral somatic delusion (OSD). OSD may be either primary (monosymptomatic) or secondary to another disease, such as depression or cerebral infarction. Although the presenting complaints of patients with primary and secondary OSD are nearly indistinguishable, symptoms in patients with secondary OSD seem to be resistant to treatment compared with those in patients with primary OSD. Moreover, right dominant cerebral blood flow (CBF) has been reported in patients with primary OSD, but the difference in CBF between patients with primary and secondary OSD remains unclear. The aim of this study was to assess the differences in clinical characteristics and CBF distribution between patients with monosymptomatic OSD (non-depression group) and OSD in conjunction with remitted depression (depression group).

**Methods:**

Participants were 27 patients of a psychosomatic dentistry clinic, all diagnosed with OSD. They were categorized into either the non-depression group (17 patients) or the depression group (10 patients) on the basis of assessments by their personal medical providers. CBF was examined using single-photon emission computed tomography.

**Results:**

There was no difference in clinical presentation between the two groups. A significant right dominant asymmetry in the temporal and posterior cerebral regions was observed in both groups. In the central region, a right dominance was seen in the non-depression group, while a left dominance was seen in the depression group. Moreover, the mean regional CBF values for patients in the depression group were significantly lower in several regions (including bilateral callosomarginal, precentral, angular, temporal, posterior cerebral, pericallosal, lenticular nucleus, thalamus, and hippocampus; and right central and cerebellum) than for patients in the non-depression group.

**Conclusion:**

These results suggest that the temporal and posterior cerebral regions are involved in in the pathophysiology of OSD, regardless of depression history, and that widespread CBF reduction is a characteristic of remitted depression.

## Background

A significant number of patients who visit dental clinics report unusual sensations in the oral area (e.g., sticky or slimy saliva, the presence of foreign objects such as sand, bubbles, eggs or metal pieces, and so on), but no corresponding abnormality can be found on physical examination. Such patients are recognized as having oral somatic delusion (OSD), oral paresthesia, or oral cenesthopathy.

Monosymptomatic OSD [1-3] is categorized as a delusional disorder, somatic type (DDST) [4], but it sometimes appears secondary to a psychiatric disorder such as schizophrenia or depression [5,6] or to cerebrovascular disease [7]. Most patients with monosymptomatic OSD first visit a dental clinic because their cenesthopathic symptoms are limited to the oral cavity, convincing them that there is a physical problem in that area. Some patients with OSD are referred to dentists by their psychiatrists because of oral cenesthopathic symptoms that developed while their depression was in remission. Because the chief complaints of patients with primary and secondary OSD are very similar, it is difficult to distinguish between them on the basis of symptoms alone. However, the symptoms of patients with secondary OSD seem to be resistant to treatment compared with those of patients with primary (monosymptomatic) OSD.

Several studies of regional cerebral blood flow (rCBF) in patients with DDST have shown left temporal and left parietal lobe hypoperfusion that normalized as the symptoms improved [8-10]. We also previously reported a case of OSD in which a rightward asymmetry of blood flow in the temporal area disappeared after successful treatment [2]. Furthermore, another of our recent studies demonstrated that the CBF in patients with OSD had a right > left asymmetry in the frontal and temporal regions compared with the CBF of control patients [3]. Thus, a rightward asymmetry, especially in the temporal area, may be associated with the development of cenesthopathy.

In the current study, we hypothesized that the clinical features and the CBF distribution patterns in secondary OSD, especially when it appears in the remitted period of depression, are different from those in primary (monosymptomatic) OSD.

With the aim of clarifying the differences between primary (monosymptomatic) OSD and secondary OSD associated with the remitted period of depression, we investigated the clinical characteristics and the CBF distributions using single photon emission computed tomography (SPECT).

## Methods

This study involved 27 patients with OSD who visited the psychosomatic dentistry clinic of Tokyo Medical and Dental University dental hospital in Tokyo, Japan. All of the subjects provided written informed consent. The exclusion criteria were the presence of a delusion or hallucination involving a body part other than the oral region, abnormalities in magnetic resonance imaging findings, a low Revised Hasegawa Dementia Scale (HDS-R) score (<25) to exclude dementia, and the presence of other neurological diseases, such as Parkinson’s disease. Because of previous reports that pain causes changes in the CBF [11-13], subjects with pain in their oral cavity or any other body parts were also excluded.

Based on the results of patient interviews and assessments by their attending doctors (including psychiatrists, family physicians and other medical specialists), the subjects were grouped as follows: non-depression group (no history of psychiatric disorders) and depression group (oral symptoms appeared during a remission period of a major depressive disorder). For the subjects in the depression group, all the subjects’ depressive states were assessed as “remitted” or “very much improved” by their psychiatrists.

The depressive state of each subject was assessed at the time of the first examination using the Zung Self-Rating Depression Scale (SDS). All subjects were right-handed except subject No. 11.

### Ethics statement

This study was conducted with the approval of the Ethical Committee of Tokyo Medical and Dental University (no. 356).

### Demographic data

The demographic data for all subjects are listed in Tables 1 and 2. The non-depression group consisted of 17 subjects (15 women, 2 men) with no history of psychiatric disorders; the mean ± standard deviation (SD) age was 67.65 ± 10.33 years (range, 50–83 years). The depression group consisted of 10 subjects (7 women, 3 men) who developed OSD during a remission period of depression; the mean ± SD age was 67.60 ± 6.59 years (range, 57–78 years).

**Table 1:** **Profiles of subjects in the non-depression group**

No.	age sex	Duration of illness (months)	Complants	Onset opportunities	Antipsychotics beeing taken at time of first examination	Antipsychotics beeing taken at the time of SPECT examination	SDS at time of first examination
1	75 F	20	too much saliva	extraction	sulpiride 50 mg, zolpidem 10 mg	aripiprazole 1.5 mg, sulpiride 50 mg, zolpidem 10 mg	43
2	72 F	9	numerous “balloons” in a hole in the palate	none	triazolam 0.125 mg	aripiprazole 1.5 mg, triazolam 0.125 mg, zolpidem 5 mg	23
3	50 F	13	bubbles and a filmy substance exuding from teeth	none	sulpiride 150 mg, clotiazepam 5 mg, lorazepam 0.5 mg	sertraline 25 mg, milnacipran 100 mg, lorazepam 1.5 mg	33
4	80 F	10	presence of something like liquid exuding from her mandibular	reflux esophagitis	-	perospirone 12 mg, sertraline 25 mg	44
5	64 M	18	presence of something like plastic ties in the space between teeth and bone	prosthesis (denture)	flunitrazepam 4 mg, nitrazepam 5 mg	sertraline 75 mg, mirtazapine 30 mg, ethyl loflazepate 2 mg	24
6	76 F	9	sticky, glue-like substance in mouth	none	-	aripiprazole 3 mg	29
7	83 F	26	garbage exuding from beneath dentures; sticky and sandy mouth	prosthesis (denture)	-	aripiprazole 1.5 mg	56
8	81 F	16	sticky mouth and dry lips	none	clonazepam 1.5 mg, zopiclone 7.5 mg	mirtazapine 7.5 mg, zopiclone 7.5 mg, clonazepam 1.5 mg	44
9	70 F	56	sticky mouth; bitter and salty substances exuding from mouth	prosthesis (denture)	etizolam 1.0 mg	mirtazapine 15 mg	64
10	60 F	21	sticky mouth; sour and bitter taste; bubbles and slimy substances persists around throat	none	etizolam 1.0 mg	sertraline 25 mg, mirtazapine 15 mg, etizolam 0.5 mg	64
11	71 M	45	lint exuding from interdentium	none	zolpidem 10 mg	mianserine 10 mg, bromazepam 4 mg, zolpidem 10 mg	38
12	70 F	23	movement of a dental implant and balls in her mouth; pins coming out through the night; sticky saliva	prosthesis (denture)	-	amitriptyline 30 mg	74
13	72 F	8	movement of a dental implant and balls in her mouth; pins coming out through the night; sticky saliva	prosthesis (denture)	-	ethyl loflazepate 1.5 mg	34
14	61 F	20	sticky mouth and too much saliva	prosthesis	-	-	50
15	55 F	22	dental bridge feels too tight	extraction	-	aripiprazole 1 mg	47
16	50 F	10	sticky mouth and salty-sweet taste	none	triazolam 0.125 mg	aripiprazole 1 mg	46
17	60 F	54	sticy mouth and salty saliva	prosthesis	-	-	33

SPECT: single-photon emission tomography.

SDS: Zung Self-Rating Depression Scale.

**Table 2:** **Profiles of subjects in the depression group**

No.	age sex	Duration of illness (months)	Duration between onset of depression and onset of oral cenesthopathy (years)	Complants	Onset opportunities	Antipsychotics beeing taken at time of first examination	Antipsychotics beeing taken at the time of SPECT examination	SDS at time of first examination	Depressive state at onset of oral cenesthopathy
18	57 F	16	1	too much saliva; feels like mouth is vacuumed.	psychological stress	amitriptyline 55 mg, flunitrazepam 2 mg	mirtazapine 15 mg, flunitrazepam 2 mg	34	remission
19	70 F	81	15	tight gums from wich salive is squeezing out	psychological stress	alprazolam 0.4 mg, brotizolam 0.25 mg, amoxapine 20 mg	aripiprazole 1.5 mg	29	very much improved
20	68 F	18	7	gum-like sticky substance persists over thin, hard substance on teeth	stomatitis	haloperidole 0.75 mg, amitriptyline 100 mg, brotizolam 0.5 mg, clotiazepam 10 mg	haloperidole 0.75 mg, aripiprazole 6 mg, amitriptyline 85 mg, brotizolam 0.25 mg, clonazepam 1.5 mg	36	very much improved
21	69 F	13	7	greasy saliva coating teeth	none	paroxetine 20 mg	paroxetine 20 mg, brotizolam 0.25 mg, trihexyphenidyl 4 mg	41	very much improved and stabilized
22	78 F	50	7	slimy liquid exudingfrom throat	none	paroxetine 15 mg, alprazolam 1.2 mg, brotizolam 0.25 mg, lormetazepam 1 mg, flunitrazepam 1 mg	mianserin 10 mg	50	remission
23	74 F	3	1	tasteless; substance resembling frog eggs or pins persists over tongue and membranes or fibers present on labial mucosa; sandy mouth	none	sertraline 50 mg	sertraline 50 mg	49	very much improved
24	70 M	60	3	dry mouth; space in palate is too full causing a chocking feeling	dental implants	triazolam 0.05 mg, etizolam 0.5 mg	etizolam 0.25 mg	36	very much improved and stabilized
25	58 M	34	6	metal substance extrudingt from teeth; too much saliva	dental implants	clomipramine 100 mg, mirtazapine 15 mg, ethyl flozepate 1 mg, etizolam 0.5 mg,	aripiprazole 3 mg, clomipramine 100 mg, mirtazapine 15 mg, etizolam 0.5 mg	30	almost remission
26	63 F	16	2	bubbly saliva	psychological stress	olanzapine 2.5 mg, fluvoxamine 75 mg, zopidem 10 mg, brotizolam 0.5 mg, lormetazepam 2 mg	aripirazole 3 mg, fluvoxamine 75 mg, ethyl loflazepate 2 mg, zolpidem 10 mg, brotizolam 0.5 mg, lormetazepam 2 mg, biperiden 1 mg	44	very much improved and stabilized
27	69 M	62	18	sticky mouth	prosthesis	zopiclone 7.5 mg, cloxazolam 3 mg	aripiprazole 1.5 mg, mirtazapine 7.5 mg	40	remission

SPECT: single-photon emission tomography.

SDS: Zung Self-Rating Depression Scale.

### Brain perfusion SPECT

SPECT imaging was performed with the subjects resting and supine, with eyes closed and in quiet surroundings after injection of a bolus of 600 MBq of Tc-99 m ethylcysteinate dimer (^99m^Tc-ECD) via the right brachial vein.

First, the passage from the heart to the brain was monitored using a rectangular large-field dual-head gamma camera (E.CAM Signature; Toshiba, Tokyo, Japan) equipped with low-energy high-resolution parallel-hole collimators. Data acquisition consisted of a sequence of 100 frames at a rate of 1 s/frame using a 128 × 128 matrix. Next, SPECT images were obtained using the same gamma camera equipped with fan-beam collimators. The energy window was set at 140 keV ± 15%, and 45 step-and shoot images were obtained throughout 180 degrees of rotation (128 × 128 matrix, 1.72 mm/pixel) with an acquisition time of 30 s/step. All the images were reconstructed using the ordered subset expectation maximization method and were smoothed three-dimensionally using a Butterworth filter. The Chang method was used to correct for gamma ray attenuation.

### Data analysis

For the regional CBF (rCBF) quantification, the Patlak plot method [14,15] was applied to the ^99m^Tc-ECD cerebral blood perfusion SPECT images to measure the mean CBF (mCBF) of bilateral cerebral hemispheres. Quantitative flow-mapping images were then obtained from the qualitative cerebral perfusion SPECT images using the Patlak plot graphical analysis and Lassen’s correction [16,17].

The rCBF quantification was performed using a three-dimensional stereotactic regions of interest template (3DSRT) program [18,19]. 3DSRT is a fully automated rCBF quantification program that can be used to examine a total of 636 regions of interest (ROIs). These 636 ROIs are categorized into 12 brain segments on the 3DSRT template: callosomarginal, precentral, central, parietal, angular, temporal, posterior cerebral, pericallosal, lenticular nucleus, thalamus, hippocampal, and cerebellar segments. The blood flow to each ROI was quantified in mL/100 g/min.

For the analysis, the rCBF values obtained from the 3DSRT data and mCBF values obtained from the Patlak Plot method were used. As an index of brain perfusion asymmetry, the right to right + left ratio [R/(R + L) ratio] was calculated for the 12 brain segments in each subject.

The R/(R + L) ratio equaled the rCBF values for the target segment on the right side divided by the sum of the rCBF values for the corresponding segments on the right and left sides.

The mean rCBF values and the mean R/(R + L) ratios for each brain segment and the mCBF values for global and for right and left hemispheres were calculated separately for the non-depression and the depression group. The results were expressed as the mean ± SD.

### Statistical analysis

PASW 17.0 software (IBM, Chicago, IL, USA) was used to perform the Mann–Whitney *U* test and the Pearson’s χ^2^ test. All the tests were two-tailed, and *P* values < 0.05 were considered statistically significant.

## Results

### Clinical features of patients in non-depression and depression groups

Tables 1 and 2 show the demographic and clinical data of the OSD patients involved in the present study. No significant differences in age (*P* = 0.639) or sex (*P* = 0.239) were observed between the non-depression and the depression groups. In our previous report [3], the subjects were also predominantly female (6 women, 2 men), but the mean ± SD age was 75.9 ± 6.0 years, which is older than the subjects in the present study.

As shown in Tables 1 and 2, the chief complaints in both groups were similar: “sticky or slimy saliva”, “foreign body sensation”, “bitter or sour taste”, or “something resembling bubbles, pieces of metal, or plastic”. The duration of illness in the depression group was 27.14 ± 20.52 months (range, 3–81 months), which was longer than that in the non-depression group (22.35 ± 15.18 months; range, 8–56 months). However, the difference was not significant (*P* = 0.309). Despite the long duration of symptoms, all the subjects continued to socialize. In the depression group, the mean time from onset of depression to onset of OSD varied (6.70 ± 5.76 years; range, 1–18 years), but all of the subjects were in the remission period of depression when the OSD appeared, according to the assessments made by their psychiatrists. In the depression group, the medications that were being taken at the time of the first examination were mainly anxiolytics or hypnotics, and some patients were not taking any antidepressant at all. Regarding the SDS score, the mean score in the non-depression group (43.9 ± 14.4) was somewhat higher than that in the depression group (38.9 ± 7.26), but the difference was not statistically significant (*P* = 0.245). No apparent difference was observed in any clinical characteristic categories between the non-depression and depression groups.

### Difference in rCBF between non-depression and depression groups

**Figure 1:**
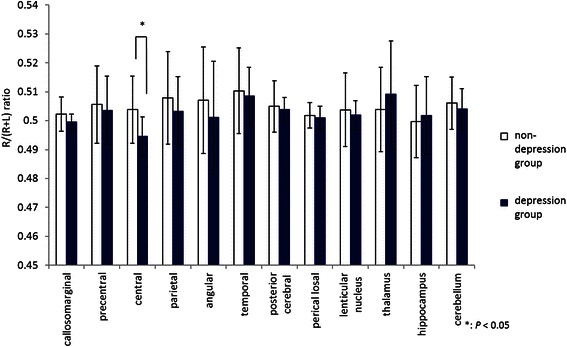
**Mean R/(R + L) ratio for each segment.** A significant difference was observed in the central: a right dominant asymmetry was observed in the non-depression group, and a left dominant asymmetry was observed in the depression group.

Table 3 shows the mean rCBF values for the 24 segments in each of the groups. In the non-depression group, a right dominant asymmetry was observed for many segments, especially the temporal, posterior cerebral, and cerebellum (where significant differences were observed). In the depression group, a significant right dominant asymmetry was observed in the temporal and posterior cerebral. Furthermore, in the depression group, the central region showed a significant left dominant asymmetry. We examined and compared the R/(R + L) ratio in each region for both groups (Figure 1). A right dominant asymmetry was observed in many regions in both the non-depression and depression groups. However, a significant difference between the non-depression and depression groups was observed only for the central region, with a right dominant asymmetry observed in the non-depression group and a left dominant asymmetry observed in the depression group.

**Table 3:** **Mean rCBF values for 24 segments in the non-depression and depression groups**

	Right hemisphere	Left hemisphere	P value
non-depression group			
callosomarginal	43.9 ± 3.82	43.56 ± 4.19	0.156
precentral	46.41 ± 4.18	45.44 ± 4.62	0.114
central	44.61 ± 3.54	43.98 ± 3.99	0.198
parietal	44.28 ± 4.03	42.94 ± 4.07	0.056
angular	46.92 ± 4.96	45.54 ± 3.90	0.097
temporal	43.74 ± 3.34	41.99 ± 3.44	0.012*
posteriorcerebral	48.34 ± 3.85	47.41 ± 3.87	0.042*
pericallosal	46.74 ± 4.16	46.43 ± 4.52	0.115
lenticular nucleus	50.13 ± 4.58	49.39 ± 4.54	0.260
thalamus	46.94 ± 5.33	46.15 ± 4.66	0.218
hippocampus	37.74 ± 4.02	37.81 ± 4.31	0.876
cerebellum	55.67 ± 5.61	54.42 ± 6.34	0.018*
depression group			
callosomarginal	39.19 ± 4.33	39.28 ± 4.40	0.564
precentral	41.78 ± 5.22	41.12 ± 4.39	0.344
central	40.71 ± 4.42	41.55 ± 4.03	0.030*
parietal	39.84 ± 5.14	39.27 ± 4.49	0.366
angular	40.71 ± 4.30	40.56 ± 4.61	0.887
temporal	38.26 ± 3.30	36.96 ± 2.83	0.027*
posteriorcerebral	44.50 ± 3.71	43.80 ± 3.46	0.016*
pericallosal	42.14 ± 4.68	41.93 ± 4.30	0.355
lenticular nucleus	45.00 ± 3.12	44.63 ± 2.74	0.229
thalamus	41.03 ± 4.35	39.58 ± 4.39	0.186
hippocampus	33.82 ± 2.51	33.56 ± 2.43	0.662
cerebellum	51.11 ± 4.21	50.35 ± 4.72	0.142

*; P < 0.05.

In the non-depression group, significant right dominant asymmetries were observed in the temporal, posterior cerebral, and cerebellum. In the depression group, a significant left dominant asymmetry was observed in the central, and significant right dominant asymmetries were observed in the temporal and posterior cerebral.

**Figure 2:**
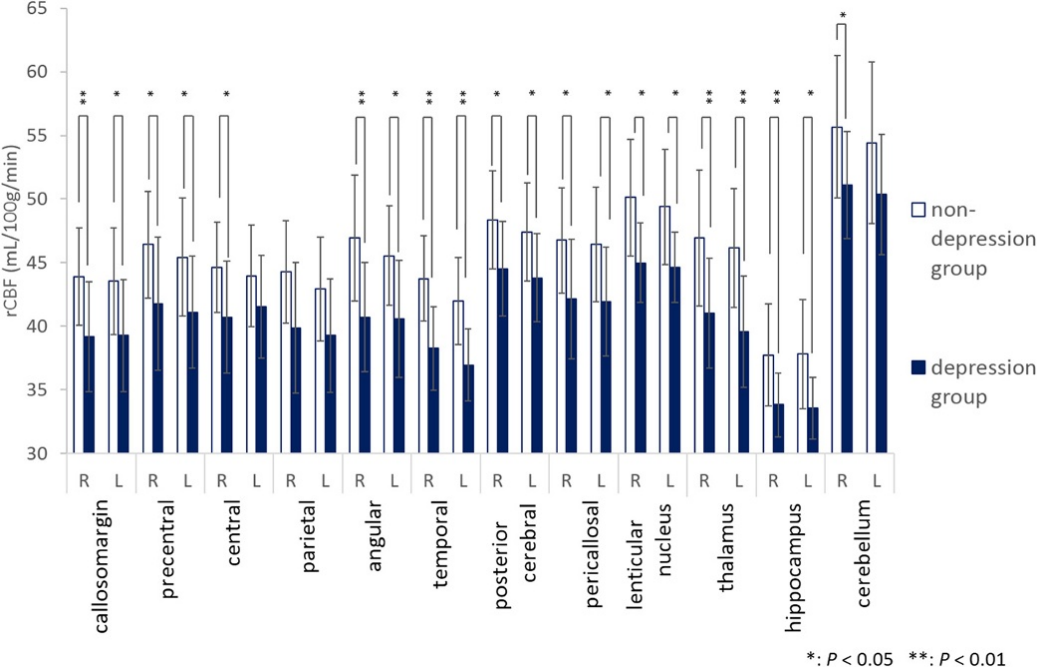
**Mean regional cerebral blood flow values for 24 segments in each group.** The mean rCBF values in the depression group were significantly lower in all segments except the left central, bilateral parietal, and cerebellum, compared with the values in the non-depression group.

**Figure 3:**
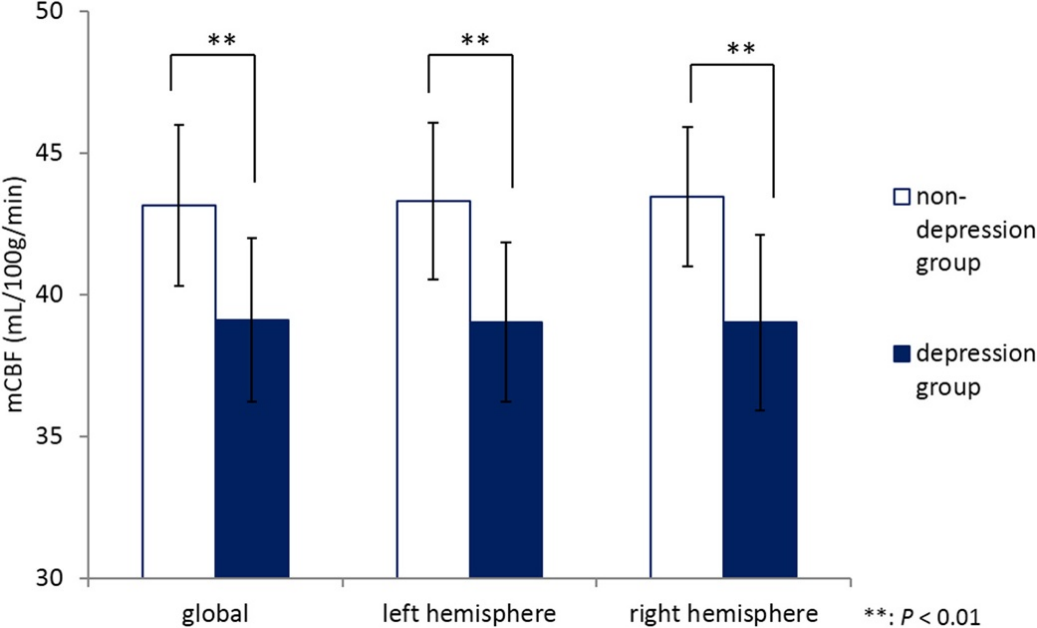
**Mean regional cerebral blood flow values for global and the left and right hemispheres.** All the mCBF values in the depression group were significantly lower than those in the non-depression group.

Figure 2 shows the difference in the mean rCBF values between the non-depression and depression groups. Analysis of each segment revealed that the mean rCBF values in the depression group were significantly lower than those of the non-depression group in the bilateral callosomarginal and precentral; right central; bilateral angular, temporal, posterior cerebral, pericallosal, lenticular nucleus, thalamus, and hippocampus; and right cerebellum. Moreover, because the rCBF patterns in both groups were similar, these reductions were not partial but were total and even. To confirm these global CBF reductions in the depression group, the mCBF values measured by the Patlak Plot method also compared the two groups. The mCBF values in the depression group were significantly lower in the global (*P* = 0.0045) and the right (*P* = 0.0014) and left (*P* = 0.0011) hemispheres than those in the non-depression group (Figure 3).

### Effects of medications on mCBF values

As listed in Table 1, 15/17 patients in the non-depression group and 10/10 patients in the depression group were taking psychotropics (antipsychotics, antidepressants, or anxiolytics) or hypnotics at the time of SPECT examination. Since the doses and types of medications vary widely, this factor is difficult to standardize. We concentrated on the use of antidepressants, which were taken by the largest number of subjects, and standardized the data using an imipramine equivalent [20] (Table 4). No significant difference in the imipramine equivalent dose was observed between the non-depression and depression groups (*P* = 0.155). The number of patients taking antidepressants was 8/17 in the non-depression group and 8/10 in the depression group. Focusing on only the patients who were taking antidepressants, the mCBF value in the depression group was significantly lower than that in the non-depression group (8 patients in each group; global *P* = 0.050; left hemisphere *P* = 0.038, right hemisphere *P* = 0.021), with no significant difference in the imipramine equivalent doses (*P* = 0.959). Within the non-depression group, the mCBF value in the 8 patients taking antidepressants was slightly lower than that in the 9 patients who were not taking antidepressants, although the difference was not significant (global *P* = 0.167; left hemisphere *P* = 0.236; right hemisphere *P* = 0.321).

**Table 4:** **Mean cerebral blood flow (mCBF) values and mean imipramine equivalent doses**

		Mean of imipramine equivalent dose	mCBF values		
			Whole brain	Left hemisphere	Right hemisphere
non-depression group	total (n = 17)	38.53 ± 65.57	43.15 ± 2.85	43.31 ± 2.78	43.45 ± 2.46
	taken antidepressants (n = 8)	81.88 ± 75.97	42.43 ± 3.59	42.60 ± 3.51	42.91 ± 3.19
	no antidepressants (n = 9)	0	43.79 ± 2.00	43.93 ± 1.93	43.93 ± 1.64
depression group	total (n = 10)	58.50 ± 51.91	39.10 ± 2.89	39.03 ± 2.82	39.02 ± 3.10
	taken antidepressants (n = 8)	73.13 ± 47.35	38.71 ± 3.03	38.70 ± 2.91	38.44 ± 3.21
	no antidepressants (n = 2)	0	40.65 ± 2.19	40.35 ± 2.76	41.35 ± 0.78

No difference in the imipramine equivalent doses was observed between the non-depression and depression groups. No difference in the mCBF values was observed between subjects taking and not taking antidepressants. However, focusing on only the patients who took antidepressants, the mCBF value in the depression group was significantly lower than that in the non-depression group.

## Discussion

The present study had two principal findings. First, no apparent clinical symptomatic difference was observed between the pure and depression groups, and a right dominant asymmetry was observed in the temporal and posterior cerebral in both groups. On the other hand, in the central, a significant difference was observed between both groups: a left dominant asymmetry was observed in the depression group, while a right dominant asymmetry was observed in the pure group.

Second, the mean rCBF values in the depression group were significantly lower than those in the pure group in many regions. Moreover, in the depression group, the mean mCBF values in the global, and the right and left hemispheres were also significantly lower than those in the pure group.

### Right dominant asymmetry in OSD

A right dominant asymmetry was observed in the non-depression group, but the asymmetry was not as prominent as that in our previous report [3]. The reason for this difference might have been the different characteristics and number of subjects in the two studies. Because the results of our previous study suggested that OSD has various subtypes, patients with cenesthopathic symptoms in other body parts or who were experiencing pain were excluded from the present study. Such careful screening to select more homogeneous OSD might have contributed to the difference in the results between the present study and our previous study. The difference in the mean age might also have contributed to the difference in the degree of asymmetry. However, a significant right dominant asymmetry in the temporal region was common to both the present and our previous studies. Our previous case report [2] showed a right dominant asymmetry in the temporal region that became less marked after an improvement in the symptoms of OSD. Therefore, the temporal region might play an important role in the pathophysiology of OSD. Moreover, since the right dominant asymmetry in the temporal region was found in not only the non-depression group but also in the depression group, the temporal region might be involved in the pathophysiology of OSD regardless of the history of depression. On the other hand, no previous report about the right dominant asymmetry in the posterior cerebral was found. Based on the results of this study, the posterior cerebral as well as the temporal region might be involved in the pathophysiology of OSD; however, further studies are necessary to confirm this theory.

In the central, a significant difference was observed between the non-depression and depression groups: right dominance was observed in the non-depression group, while left dominance was observed in the depression group. This result suggests that the central region, including the central sulcus, which is well known as a somatosensory and motor area, is involved in the pathophysiology of OSD. Further investigations are expected.

### Global reduction in CBF in the depression group

Mean rCBF values of the depression group were significantly lower than those of the non-depression group in several regions, and the mCBF values were also significantly lower in the global and the right and left hemispheres.

Whether the non-depression group or the depression group showed abnormal perfusion is unknown, since they were not compared with non-OSD controls. However, in our previous study [3], the absolute CBF values in subjects with OSD (8 cases) were somewhat higher than those of subjects without OSD (8 cases), although the difference was not significant. Therefore, a global CBF decrease in the depression group may be a more likely explanation for the differences than a CBF increase in the non-depression group.

The reasons for these CBF reductions should be carefully examined from several aspects.

### Effects of medications on mCBF values

The first reason that should be considered is the effect of medications. Some medications, including antidepressants, antipsychotics, anxiolytics, and hypnotics, reportedly decrease or increase the CBF [21-23]. However, the results in this study suggest that the effect of medications is not sufficient to explain the CBF reduction observed in widespread regions in the depression group, even when the data is partially corrected for medication use.

### Effects of depression itself on the mCBF values

Another reason that should be considered is the effect of depression itself. The theory that CBF may be widely decreased by of comorbid depression is fascinating. There are many previous studies of CBF in depressive patients. Some reported a total reduction [24-26], which is similar to the results of the present study, but others reported regional reductions in the frontal, temporal, or parietal regions [27-29]. Because of such varied and inconsistent results, CBF changes in depression are still unclear. On the other hand, remitted depression has also been studied with regard to several factors including CBF, and various differences between subjects with and without depression have been found, even though the subjects with depression were assessed as being clinically remitted [30-32].

In the present study, the subjects in the depression group were assessed as having improved or remitted depression based on referral letters from their psychiatrists rather than on rigorous standards of remission in depression [33,34]. However, the small doses of medication that were being taken and the relatively low SDS scores suggest that subjects in the depression group would likely be categorized as having strictly defined remitted depression.

Taken together, the widespread reduction in CBF observed in the depression group in the present study might reflect the vulnerability of subjects with remitted or almost-remitted depression.

There were three limitations of the present study. The first was that almost all subjects were taking medications at the time of SPECT examination. No significant difference was found in the imipramine equivalent doses; however, other medications (such as antipsychotics, anxiolytics, or hypnotics) and their interactions could affect the CBF. The second limitation was that the depressive states of subjects were not assessed by rigorous standards of remission in depression. The third limitation was the small number of subjects, especially in the depression group. To examine statistical significance with adequate dependability, more studies with a larger number of subjects are necessary.

Regardless of the careful screening to select more homogeneous OSD, subjects of this study still exhibited a range of clinical characteristics and CBF distribution patterns. Further studies using more subjects from many different clinical aspects are needed to clarify the diversity and pathophysiology of OSD.

## Conclusions

In conclusion, in the present study, no clinical symptomatic difference was observed, and a significant right dominant asymmetry in the temporal and posterior cerebral regions was found in subjects both with monosymptomatic (primary) OSD and with OSD associated with remitted depression. Moreover, CBF in the depression group was significantly decreased in widespread regions, compared with that in the non-depression group. This phenomenon might reflect medication use, but it is more likely that widespread CBF reduction is a characteristic of remitted depression.

To clarify the pathophysiology of OSD, further studies using more subjects and details are needed.

## Abbreviations

CBF: Cerebral blood flow
DDST: Delusional disorder, somatic type
mCBF: Mean cerebral blood flow
OSD: Oral somatic delusion
rCBF: Regional cerebral blood flow
ROI: Region of interest
SPECT: Single-photon emission computed tomography
HDS-R: Revised hasegawa dementia scale
^99m^Tc-ECD: Tc-99 m ethylcysteinate dimer
3DSRT: Three-dimensional stereotactic regions of interest template
SDS: Zung self-rating depression scale
